# Supplementary material for: Extracellular Vesicles Derived from Kefir Grain Lactobacillus Ameliorate Intestinal Inflammation via Regulation of Proinflammatory Pathway and Tight Junction Integrity
Source: Biomedicines. 2020 Nov 20;8(11):522. doi: 10.3390/biomedicines8110522 (PMC7709018; doi:10.3390/biomedicines8110522)
Supplement: Supplementary file 1 [file biomedicines-08-00522-s001.pdf]

Supplementary Materials

Table S1: List of RT-PCR primers used in this study.

Gene	Forward primer	Reverse primer
IL-2	AACTCCTGTCTTGCATTGCAC	GCTCCAGTTGTAGCTGTGTTT
IL-8	ATAAAGACATACTCCAAACCTTTCCAC	AAGCTTTACAATAATTTCTGTGTTGGC
TNF-α	CTTCTCCTTCCTGATCGTGG	GCTGGTTATCTCTCAGCTCCA
IL-17A	GGCTGACCCCTAAGAAACCC	AAGCAGTTTGGGACCCCTTT
IFN-γ	GCCAAGACTGTGATTGCGGG	GTCACTGCAGCTCTGAATGTTTCT
GAPDH	TGGGCTCCAAGCAGATGC	GGCTTCGCTGGCTCCCAC
β-actin	AGAGCTACGAGCTGCCTGAC	AGCACTGTGTTGGCGTACAG
